# Sample size calculation for data reliability and diagnostic performance: a go-to review

**DOI:** 10.1186/s41747-024-00474-w

**Published:** 2024-07-05

**Authors:** Caterina Beatrice Monti, Federico Ambrogi, Francesco Sardanelli

**Affiliations:** 1 Postgraduation School in Radiodiagnostics, University of Milan, Milan, Italy; 2 Department of Clinical Sciences and Community Health, University of Milan, Milan, Italy; 3 IRCCS Policlinico San Donato, San Donato Milanese, Milan, Italy; 4 Present Address: Lega Italiana per la lotta contro i tumori (LILT) Milano Monza Brianza, Milan, Italy

**Keywords:** Data science, Reproducibility of results, ROC curve, Sample size, Sensitivity and specificity

## Abstract

Sample size, namely the number of subjects that should be included in a study to reach the desired endpoint and statistical power, is a fundamental concept of scientific research. Indeed, sample size must be planned *a priori*, and tailored to the main endpoint of the study, to avoid including too many subjects, thus possibly exposing them to additional risks while also wasting time and resources, or too few subjects, failing to reach the desired purpose. We offer a simple, go-to review of methods for sample size calculation for studies concerning data reliability (repeatability/reproducibility) and diagnostic performance. For studies concerning data reliability, we considered Cohen’s *κ* or intraclass correlation coefficient (ICC) for hypothesis testing, estimation of Cohen’s *κ* or ICC, and Bland-Altman analyses. With regards to diagnostic performance, we considered accuracy or sensitivity/specificity *versus* reference standards, the comparison of diagnostic performances, and the comparisons of areas under the receiver operating characteristics curve. Finally, we considered the special cases of dropouts or retrospective case exclusions, multiple endpoints, lack of prior data estimates, and the selection of unusual thresholds for α and β errors. For the most frequent cases, we provide example of software freely available on the Internet.

**Relevance statement** Sample size calculation is a fundamental factor influencing the quality of studies on repeatability/reproducibility and diagnostic performance in radiology.

**Key points**

• Sample size is a concept related to precision and statistical power.

• It has ethical implications, especially when patients are exposed to risks.

• Sample size should always be calculated before starting a study.

• This review offers simple, go-to methods for sample size calculations.

**Graphical Abstract:**

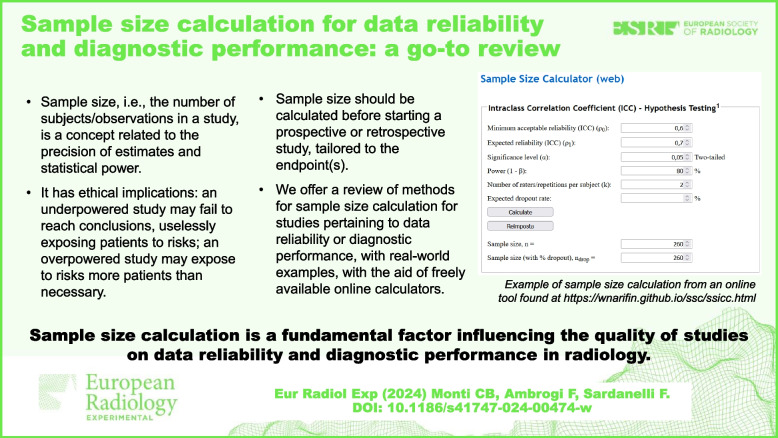

## Background

Sample size is a simple concept: it represents the number of subjects or observations in a study. Planning a proper sample size is crucial, as it is closely related to the precision of estimates and statistical power [1, 2]. This action has ethical implications, as an underpowered study may fail to reach any conclusions, uselessly exposing patients to risks, while an overpowered study may expose more patients to risk than necessary or waste human and economic resources. Moreover, sometimes in clinical research, practical issues such as patients or data availability, or other external constraints, such as time, might limit the sample size of a study.

In an ideal world, the sample size should be always calculated *a priori*, during the early stages of work planning, to determine how many data points ought to be included or retrieved [3]. Unfortunately, this is not always the case. When statistically significant differences are observed, the lack of preliminary sample size calculation remains hidden in a shadow cone. However, when differences considered clinically relevant appear to be not or merely borderline statistically significant, the lack of a preliminary sample size calculation represents a key issue which could undermine the results of the study.

In addition, it is of utmost importance for sample size to be tailored to the study endpoint(s) and be properly calculated, as poor estimations may lead to biased or unreliable results and potentially false conclusions [4]. Unfortunately, sample size calculations may prove difficult or cumbersome, especially in the clinical setting when outcome variability may be hard to predict, and prior studies may present sources of bias, which could in turn hinder such assessments [5].

To each endpoint, there is no fixed sample size in a *one size fits all* fashion. Rather, sample size should be tailored to finding an optimal balance between data availability, statistical power, and results precision. Indeed, the main factors related to sample size calculation are:

- the desired *α* error threshold, namely the highest acceptable likelihood of rejecting the null hypothesis when true;
- the desired statistical power, 1 - *β*, related to the *β* error, namely the highest acceptable likelihood of rejecting the experimental hypothesis when true;
- specific parameters related to each individual outcome [6, 7].

Of note, sample size ought to be tailored to the main endpoint of the study, with the need to verify its power for further secondary, or exploratory analyses.

A basic differentiation must be considered, distinguishing interventional from diagnostic radiology. Interventional radiology ought to be regarded as a field of therapeutic medicine, following the classic scheme applied to drug development (phases 1 to 4), with randomised controlled trials at the top of the evidence pyramid for primary/unfiltered evidence [8]. Concerning the field of diagnostic radiology, a relevant number of scientific works is either related to evaluating the reliability of imaging evaluations, or to assessing the diagnostic performance of various imaging techniques compared to diverse reference standards [9].

Reliability mainly includes *repeatability* and *reproducibility* analyses [2]: the former relating to the variability which stems from using an individual instrument and an individual reader, the latter to the overall reproducibility of an experiment with different instruments and/or readers. Planning an adequate sample size is crucial when introducing new imaging modalities, techniques, or ways of reading images, as well as to establish the basis for further studies [10]. Diagnostic performance mainly includes the assessment of overall accuracy, sensitivity, and specificity as well as area under the receiver operating characteristics curve (AUROC) [11]. Of note, both positive and negative predictive values can be considered as metrics of diagnostic performance. However, their strong dependence on prevalence may render them less useful for evaluating the intrinsic performance of a diagnostic test [8]. It is always important to address whether outcomes are related to a given measure, aiming to either a specific confidence interval or a comparison with a reference standard, or to a comparison between two distributions or samples. A proper sample size calculation represents one of the main statistical pitfalls of studies concerning diagnostic radiology [12].

Hence, the aim of this work is to offer a simple, “go-to” review of some methods for sample size calculation related to data reliability and diagnostic performance, along with online calculators and practical examples, to ease the way for those approaching such issue in a hands-on fashion. For cases of most frequent use, we provide examples of the use of software freely available on the Internet.

## Sample size for reliability

The reliability of measured variables such as size or volume of organs or lesions, physiopathological parameters such as cardiac ejection fraction, blood flow velocity, etc., or diagnostic category attribution using a “RADS” framework, is a relevant preliminary condition to confer clinical value to study results pertaining to diagnostic radiology [13].

Conveniently, the two pillars of reliability explained above (repeatability and reproducibility) may be treated the same way from a mathematical point of view. As such, studies presenting results proposed for application in clinical practice should include a Subsection, an Appendix, or a paragraph included among supplemental materials, reporting information on data reliability. Often, when this information is not included in the original submission, reviewers may ask for clarification with regards to sample size calculation, especially in the case of manuscripts proposing innovative approaches in medical imaging. These variations can be evaluated with different statistical tests with regards to the type of analysed variable (*i.e.*, categorical, discrete, or continuous), and the desired endpoint(s). In particular, the reliability of categorical variables is often reported with Cohen’s *κ*, whereas the reliability of continuous variables is usually evaluated with intraclass correlation coefficients (ICC) or Bland-Altman analyses [14]. ICC can also be used to assess agreement among more than two raters/methods.

### Cohen’s *κ* for hypothesis testing

We describe two ways for estimating the required sample size for a study using Cohen’s *κ* as a measure of reliability with reference to an online sample size calculator [15], which adopts the approaches proposed by Donner et al. [16] and Shoukri et al. [17], respectively. The first method is based on hypothesis testing and assumes a null hypothesis H_0_: *κ* = *κ*_0_ against the alternative H_1_: *κ* $\ne$ *κ*_0_ (for a two-tailed test). To calculate a sample size, it is necessary to fix one specific alternative, the expected *κ*_1_. The larger the difference between *κ*_1_ and *κ*_0,_ the smaller the sample size needed. Such a method therefore requires:

 − minimum acceptable *κ*, *κ*_*0*_;
 − the expected *κ* greater than *κ*_*0*_, *κ*_*1*_;
 − significance level, or desired α error;
 − power, or desired *β* error; and
 − proportion of outcomes, *π*.

#### Example 1

**Fig. 1:**
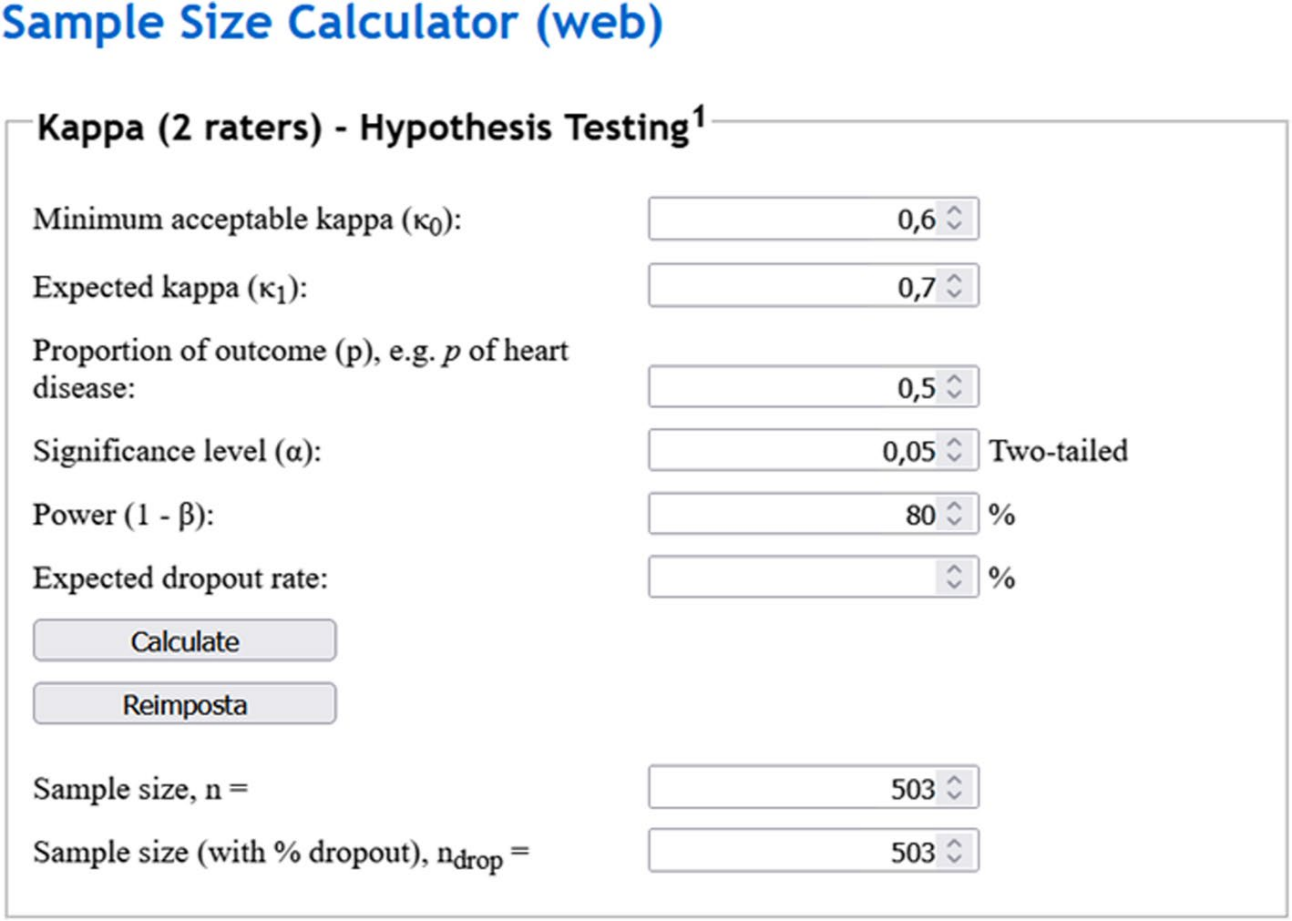
Example of sample size calculation for hypothesis testing with Cohen’s *κ*

One study assessing the reproducibility of a semiquantitative scoring system (with scores that are either 1 or 0) between two readers, aims to assess Cohen’s *κ* with a minimum acceptable *κ*, *κ*_*0*_ = 0.60, an expected *κ*_*1*_ = 0.70, an *α* error of 0.05, a statistical power of 0.80 (*β* of 0.20), and a proportion of outcomes equal to 0.5. Inserting these data into the calculator, 503 patients would be needed for the analysis. A simple tool to calculate sample size in this setting can be found online [15], as depicted in Fig. 1.

### Estimation of Cohen’s *κ*

The second method is based on fixing the precision of estimation of the Cohen’s *κ*, *i.e.*, the width of its (1-* α*) % confidence interval. To estimate the required sample size, one needs:

 − expected *κ* value, *κ*_*1*_;
 − confidence level 100(1 - α)% (generally 95%);
 − desired precision, *i.e*., the width of the (1 - α)% confidence interval; and
 − proportion of outcomes, *π*.

#### Example 2

**Fig. 2:**
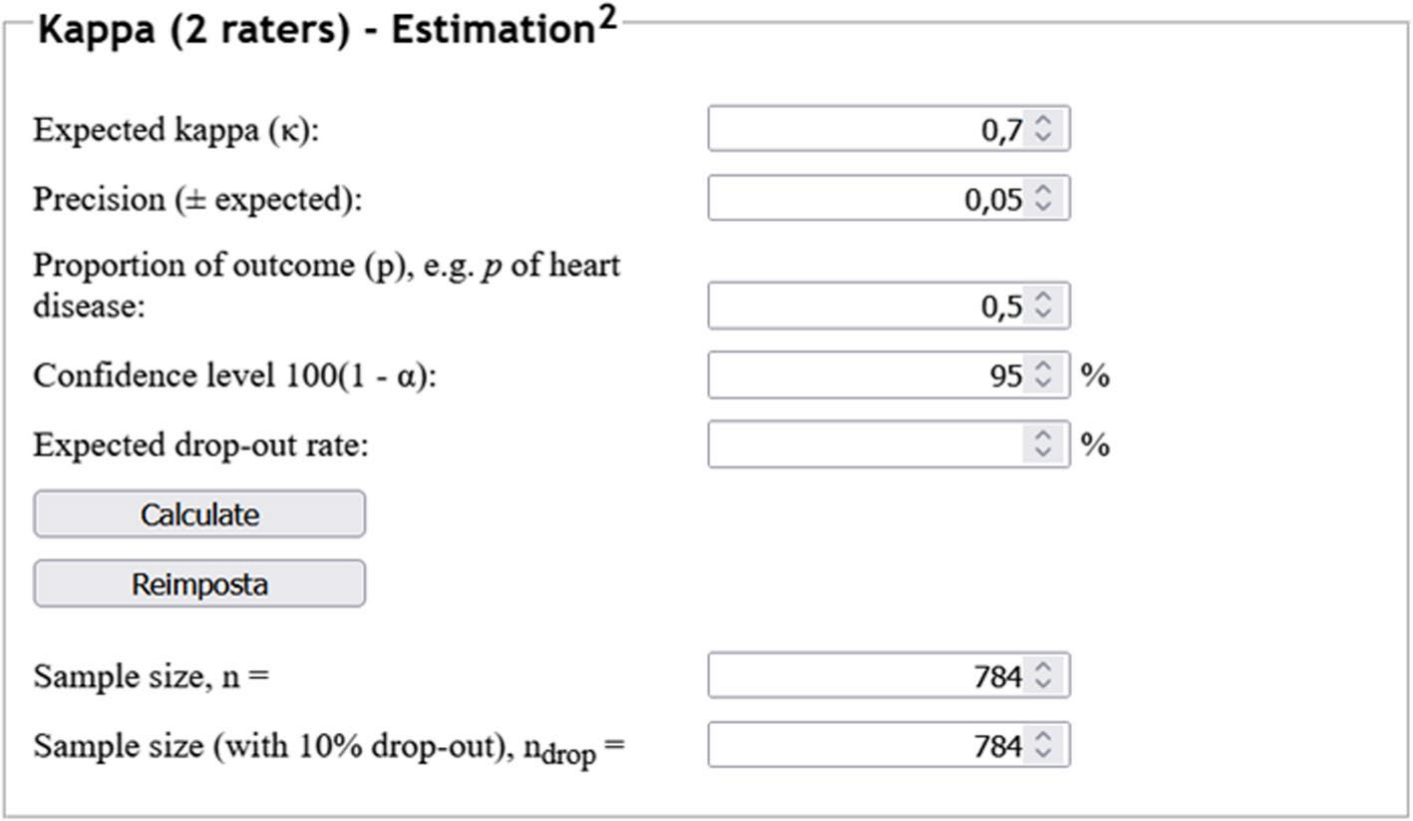
Example of sample size calculation for Cohen’s *κ* estimation

A study aims to review the reproducibility of the previously mentioned semiquantitative scoring system, basing its assumptions on a previous work reporting an expected value for Cohen’s *κ*. As such, when assuming an expected *κ* value of 0.70, a desired precision of 0.05, a confidence level of 0.95, and a proportion of outcomes equal to 0.5, the desired number of included observations for this endpoint would amount to 784, as depicted in Fig. 2.

### ICC for hypothesis testing

Taking as reference the online sample size calculator [18], which adopts the approach of Walter et al. [19] and Bonnett [20], two ways for estimating the required sample size for a study using the ICC as measure of reliability can be used. ICC is typically used when *n* subjects are evaluated by *k* raters. The first approach, based on hypothesis testing, assumes a null hypothesis, H_0_: $\rho$ = $\rho$_0_, where $\rho$ represents the correlation coefficient, or ICC value. Here the null hypothesis is not $\rho$ = 0, as this is implausible, but set to a minimum acceptable ICC, above which the expected ICC should be found. The alternative hypothesis is H_1_: $\rho \ne \rho$_0_. For sample size calculation it is necessary to specify one alternative hypothesis, $\rho$_1_, indicated by the calculator as the expected ICC.

Such method therefore requires:

 − minimum acceptable ICC, $\rho$_*0*_;
 − expected ICC greater than $\rho$_*0*_, $\rho$_*1*_;
 − significance level, or desired α error;
 − power, or desired *β* error;
 − number of raters or repetitions per subject, *k*.

#### Example 3

**Fig. 3:**
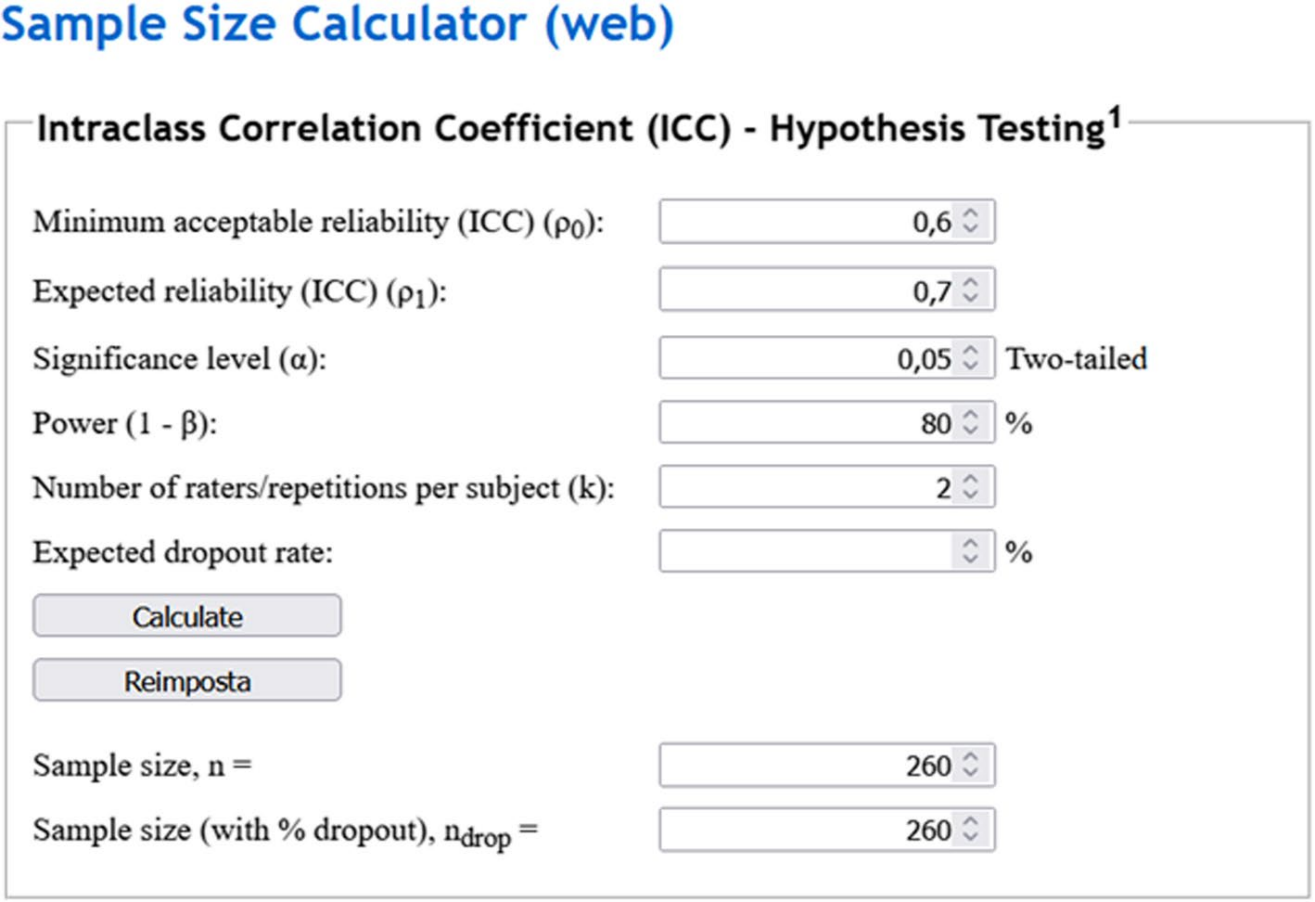
Example of sample size calculation for hypothesis testing with intraclass correlation coefficients

A study aims to appraise the reproducibility between two raters measuring a continuous variable, with hypothesis testing using ICC, for instance splenic volume in patients with haematologic malignancies. Given a minimum acceptable ICC of 0.60, an expected ICC greater than *r*_*0*_ of 0.70, an *α* error of 0.05, a statistical power of 0.80 pertaining (*β* of 0.20), and two sets of observations, using the calculator for ICC hypothesis testing sample size, the required number would result in 260 observations, as depicted in Fig. 3.

### Estimation of ICC

The online calculator offers the possibility to calculate the sample size specifying the precision of the estimate of ICC. In this case, there is no need to specify a null and alternative hypothesis, but simply, to estimate the required sample size, one needs:

 − expected ICC, *r*;
 − confidence level, (1 - α)%;
 − desired precision, *d*; and
 − number of raters or repetitions per subject, *k*.

#### Example 4

**Fig. 4:**
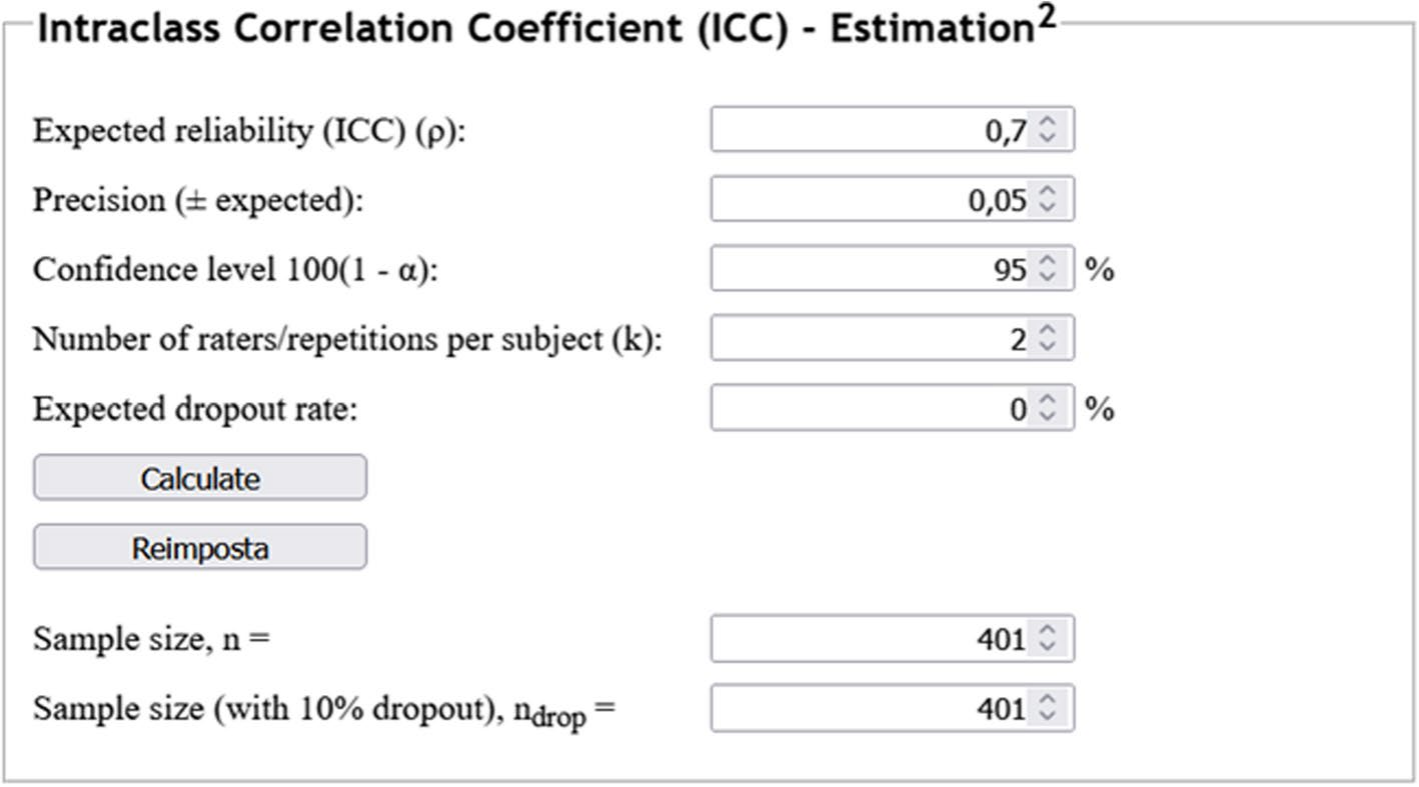
Example of sample size calculation for intraclass correlation coefficients estimation

A study aims to assess the reproducibility of splenic volume measurements in patients with haematologic malignancies between two readers, and a previous study reporting an ICC estimate is available. Assuming an expected ICC value of 0.70, a confidence level (1 - α)% = 95%, a precision of 0.05, and two sets of observations, the desired number of included observations for this endpoint would amount to 401, as depicted in Fig. 4.

### Bland–Altman analysis

A simple method to calculate the sample size required to perform a Bland-Altman analysis is described by Martin Bland himself on a *frequently asked question* section on his web page about “Design and Analysis of measurement studies” [21]. Bland proposes to consider the precision of the estimate of the 95% limits of agreement. For the calculation it is necessary to know the standard deviation of the differences between measurements of the two methods under comparison, *s*. The formula, considering a 95% confidence interval of the limits of agreement, is the following:

1 $$n=3 {\left(\frac{2* \mathrm{1,96}* s}{lw}\right)}^{2}$$

where *n* is the number of needed subjects (*i.e.*, the sample size), *s* is the standard deviation of the differences between measurements of the two methods under comparison, and *w* is the width of the 95% confidence interval. Thus, a confidence interval with half-length equal to *s* will require 12 patients while 100 patients, as suggested by Bland, will be sufficient for a half-length equal to 0.34*s*. A more elaborated approach is reported by Lu et al. [22]. A software code to calculate such sample size using an open source software, *R* [23], is reported online [24].

## Sample size for diagnostic performance

The assessment of diagnostic accuracy is perhaps the most common endpoint for studies in the field of diagnostic imaging. In this setting, accuracy could be compared to a reference standard, for example histopathology. Furthermore, sample size calculations could also be based on individual parameters, namely sensitivity and specificity, to address more specific study aims.

Of note, the choice of the reference standard can strongly influence the study results. In this sense, one can think that, as histopathological analysis is more reliable than other imaging tests, using histopathology as reference standard is the best way to run. However, this is not necessarily true, not only because histopathology may not yield comprehensive negative results (with the exception of lesion-by-lesion analysis of explanted organs or mastectomies), but also because a “hard” reference standard can introduce a bias in the selection of cases. Indeed, this way only those cases referred for biopsy or surgery are included, excluding benign lesions identified at imaging and, as such, not referred for further assessment (*spectrum bias*). This might explain why an apparently weaker reference standard, such as a negative 2-year follow-up, is used for negative readings of screening mammography, leading to a combined general reference standard (histopathology for positive cases and follow-up for negative cases) [25], which is generally used for oncology. The relevance of this aspect is well shown by the history of magnetic resonance imaging of the breast, where the first reports from the Nineties that included only few cases subsequently sent to biopsy paved the way for the *mantra* about the “low specificity” of this technique, an adverse prejudice that has persisted for a couple of decades [26]. Indeed, the choice of a proper reference standard is pivotal when calculating sample size, as the diagnostic performance of such test ought to be well known and high enough, and potential sources of bias which should be considered during study planning should be foreseen.

Moreover, no reference standard is perfect, as even the reproducibility of results from histopathology can be suboptimal [27]. This justifies – in our opinion – the use of the term “reference standard” instead of “gold standard” for any comparison in diagnostic medicine. The analysis of diagnostic accuracy, as well as that of sensitivity and specificity, implies a dichotomous evaluation by the test under consideration and the reference standard, where uncertain or intermediate cases are excluded on both sides. Such numbers should still be reported in the study results and may represent a study limitation [2].

In the following subchapters, we consider the cases of accuracy *versus* reference standard, sensitivity/specificity *versus* reference standard, comparison of different accuracies, comparison of different sensitivities/specificities and comparison of AUROCs.

### Accuracy *versus* reference standard

When calculating the sample size (*n*) for an overall accuracy study with a binary outcome *versus* a reference standard with a theoretical 100% accuracy, the necessary information is [28]:

 − level of confidence, (1 - α)%;
 − expected accuracy of the proposed method, which can be estimated from previous literature, whenever present; it can be indicated as *p*, namely the probability for the method to provide a right prediction (note that this *p* is not the *p* from the *p*-value); and
 − the acceptable margin of error, *E*, related to the half-width of the (1 - α)% confidence interval.

Subsequently, a sample size formula for the estimation of proportions might be used. A common example could be normal approximation to the binomial distribution [29]:

2 $$n=\frac{Z^2\cdot p\cdot\left(1-p\right)}{E^2}$$

Where *n* is the sample size, *Z* the z-score corresponding to the desired *α* [30], *p* the expected accuracy of the proposed method, and *E* half the width of its confidence interval. Remember that the approximation is reasonable when the total number of right predictions and the total number of wrong predictions are large (at least larger than 5).

Note that overall accuracy could be a poor indicator of diagnostic performance due to the unknown balance between sensitivity and specificity, so that of two tests with same intermediate accuracy, one may have high sensitivity and low specificity and the other one low sensitivity and high specificity. Overall accuracy alone is not informative about the real performance of a test, with the only theoretical exception of the case of 100% accuracy [2].

#### Example 5

**Fig. 5:**
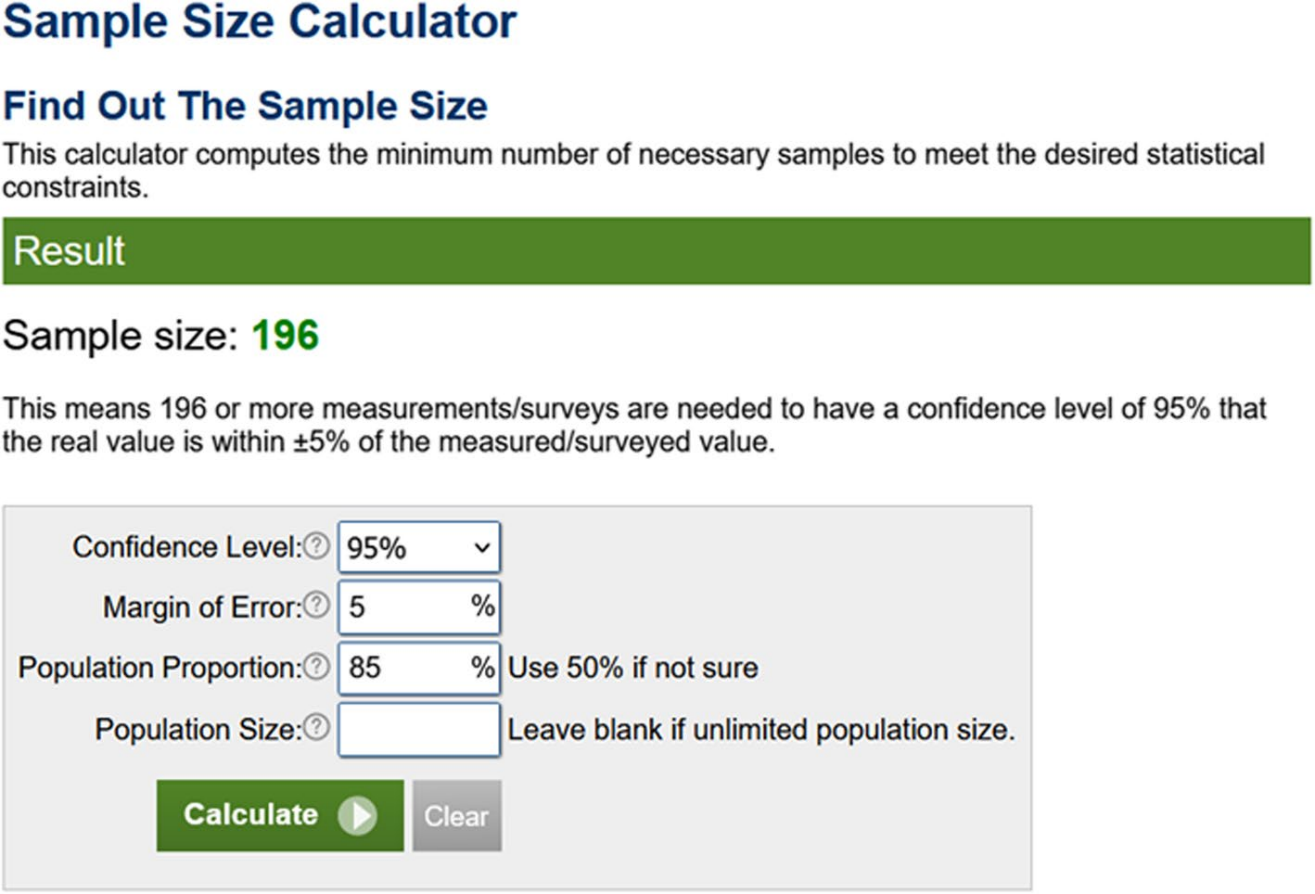
Example of sample size calculation with a specific accuracy value compared to a perfect reference standard

A study aims to review the diagnostic accuracy of an automated method for the detection of fractures on x-ray images, using computed tomography as a reference standard, with a 95% confidence level (corresponding to a Z value of 1.96); an expected accuracy (or proportion of right cases) *p*, of 0.85 (or 85%), and a margin of error *E*, equal to 0.05. The sample size calculation would indicate an *n* of 196 patients per Eq. 2. A simple tool for this type of sample size calculation can be found online [31], as depicted in Fig. 5. Another tool may also be used for such purpose [32].

Of note, in observational studies sample size is often determined by feasibility or economic reasons. In this case it is possible to use the calculator to calculate what will be the margin of error in correspondence of a specific sample size.

### Sensitivity/specificity *versus* a reference standard

When sensitivity or specificity are the main endpoints under consideration and the overall prevalence *P* is known [33], considering that:

3 $$n=\frac{{n}_{{\text{cases}}}}{P}$$

and subsequently:

4 $$n=\frac{{n}_{{\text{controls}}}}{1-P}$$

hence, the sample size starting from sensitivity (*Sens*), or specificity (*Spec*) can be calculated as follows:

5 $$n_{Sens}=\frac{Z^2\cdot Sens\cdot\left(1-Sens\right)}{E^2\cdot P}$$

6 $$n_{Spec}=\frac{Z^2\cdot Spec\cdot\left(1-Spec\right)}{E^2\cdot(1-P)}$$

where *E* is the margin of error.

#### Example 6

**Fig. 6:**
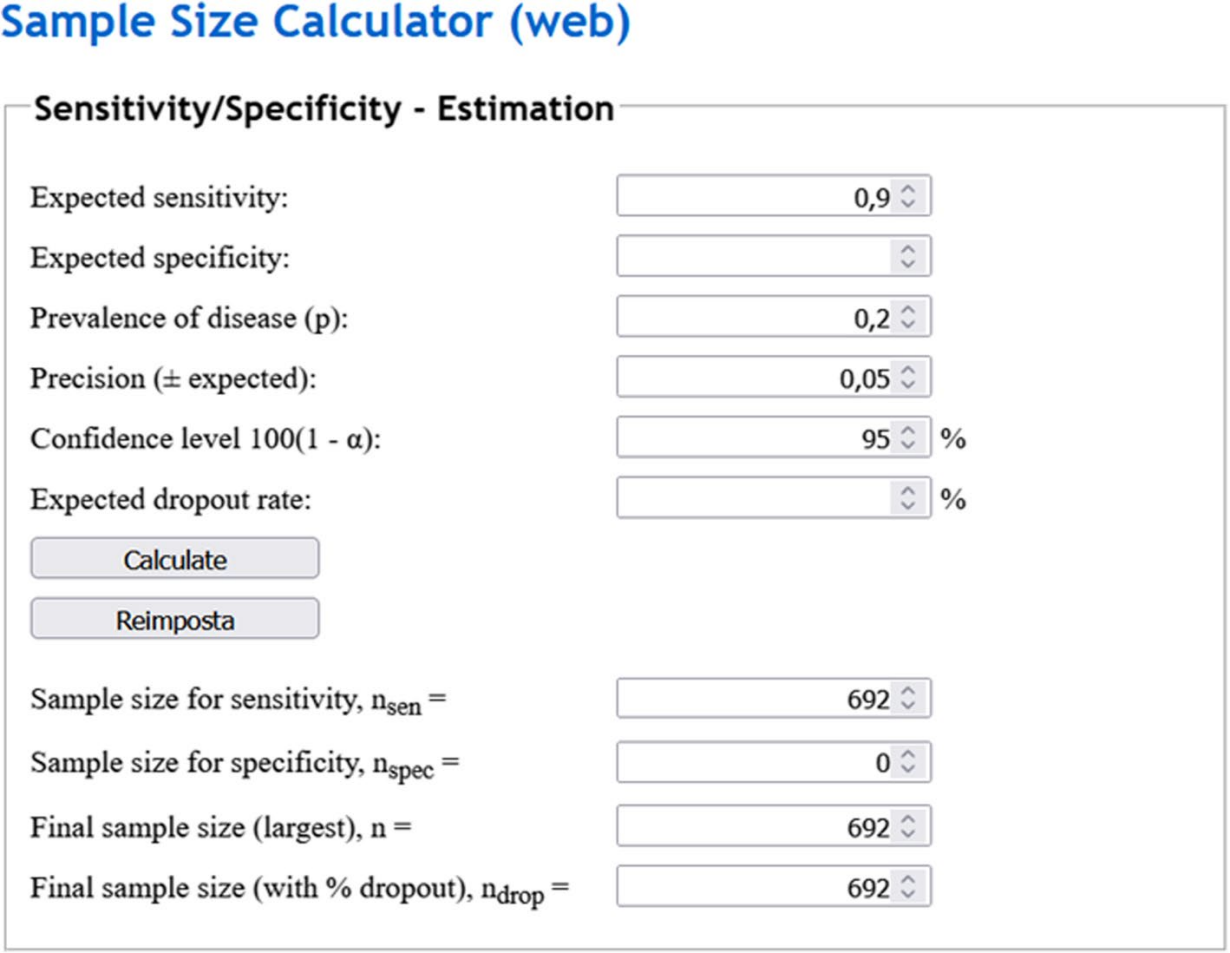
Example of sample size calculation using sensitivity as the main endpoint

We aim to assess the sensitivity of the same automated method from Example 1 for detecting fractures on x-ray using computed tomography as a reference standard, assuming a confidence level of (1 - α)% = 95%, leading to *Z* = 1.96, an expected sensitivity of 90% or 0.90, a disease prevalence *p* of 20% or 0.20, and a margin of error *E* equal to 0.05. Sample size calculations would lead to an *n* of 692 patients as per Eq. 3. A tool for calculating sample sizes using sensitivity or specificity as endpoint can be found online [34], as depicted in Fig. 6.

### Comparison of diagnostic performances

When calculating the sample size to compare the diagnostic performances (accuracy, sensitivity, or specificity) of two different methods, one important point regards the study design. The comparative study design can be unpaired, *i.e.*, study participants are assigned (ideally randomly) to one of the two tests under comparison, or paired, when both tests are performed on all subjects. For the unpaired design, the sample size can be calculated considering the statistical test for comparing independent proportions (see for example the website [35]), while for the paired design a test for dependent proportions must be considered (McNemar test [36]). In general, the following information is needed:

 − significance level *α*;
 − desired statistical power, or (1 - β); and
 − expected accuracy/sensitivity/specificity proportions, *π*_1_ and *π*_2_. In the paired design, the two proportions are called “before” and “after”, meaning that the two proportions represent the result of different tests on the same subjects.

#### Example 7

**Fig. 7:**
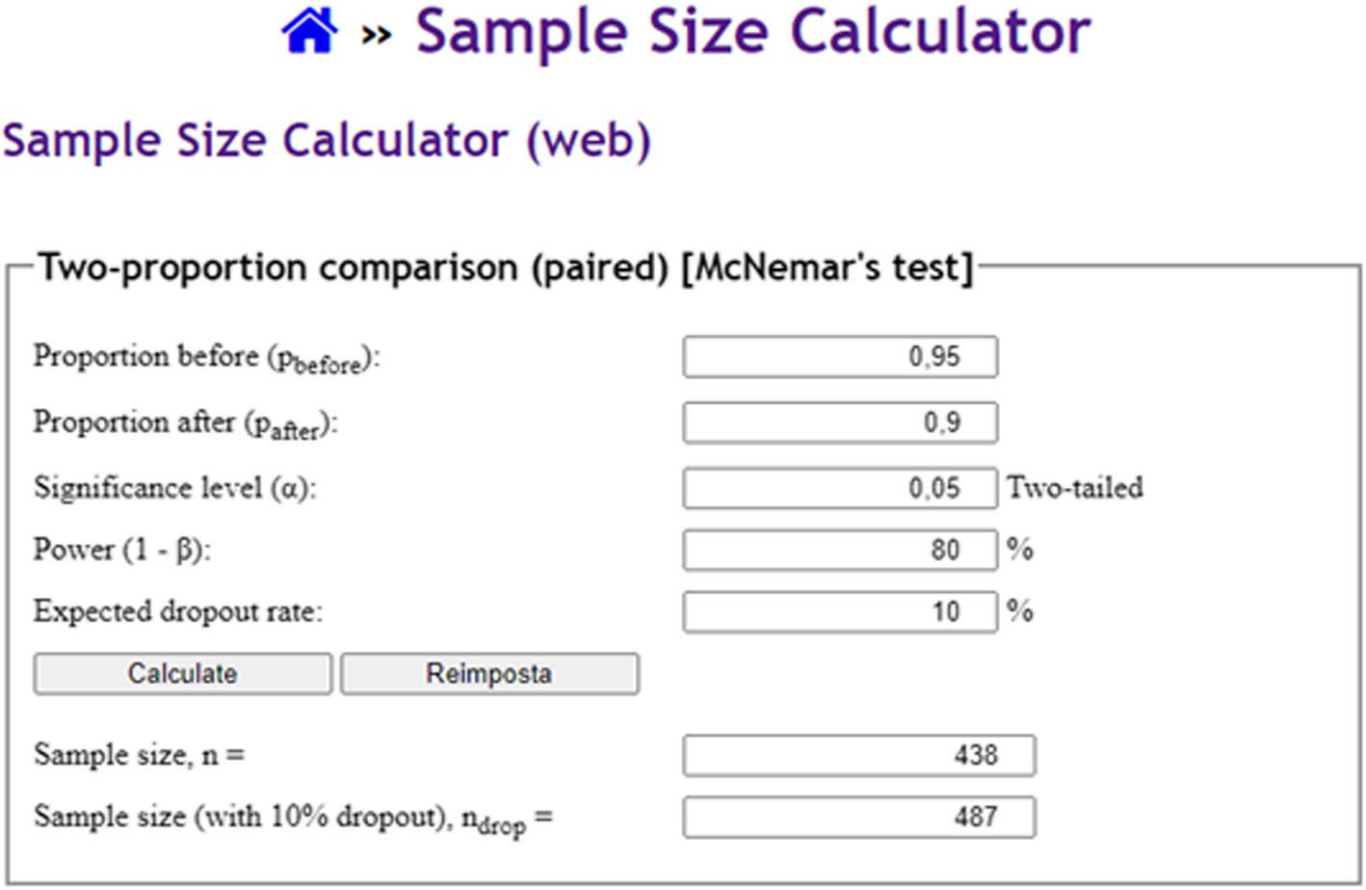
Example of sample size calculation according to a paired design comparing diagnostic accuracy

**Fig. 8:**
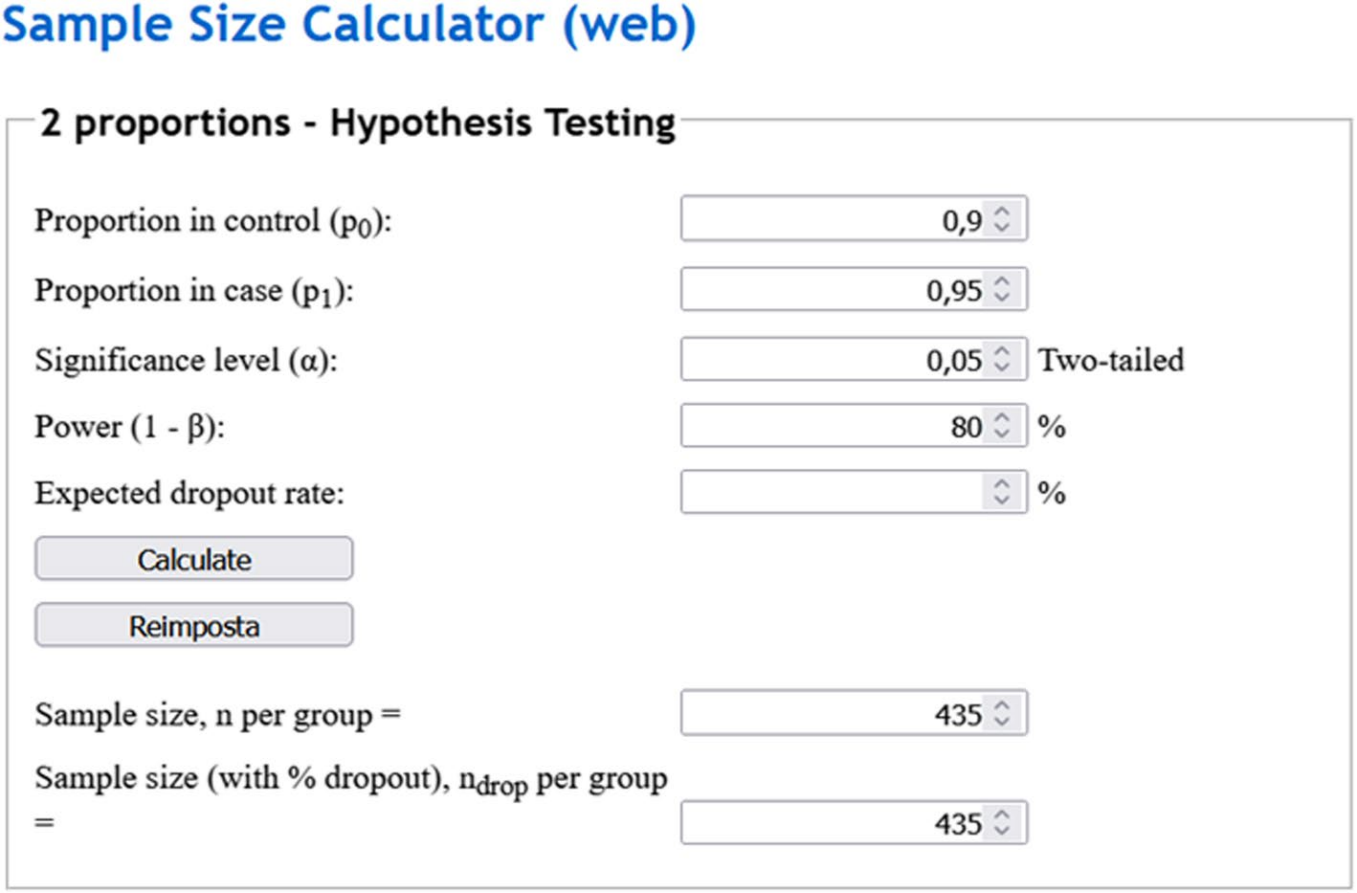
Example of sample size calculation for comparing two different diagnostic accuracies

Our endpoint is represented by the comparison of diagnostic accuracy by a proposed automatic method for diagnosing bone fractures at x-ray *versus* a human reader (radiologist), assuming an *α* error of 0.05, a power of 80%, an accuracy of 95% for the automated method, and an accuracy of 90% for the radiologist. Sample size calculations according to a paired design would lead to an *n* of 438 patients [36], as depicted in Fig. 7. Conversely, in an unpaired design this would result in n of 435 patients for each of the two groups. The tool used for computing the desired sample size is online [37], as depicted in Fig. 8. The same procedure may be used to compare sensitivity and specificity values from different diagnostic tests.

### Estimate or comparison of AUROCs

Diagnostic tests can be evaluated or compared in terms of AUROC [38]. Of note, receiver operating characteristics analysis, using all the potential reading thresholds, yields the great advantage of not being dependent on an individual threshold as instead happens for accuracy, sensitivity, specificity, and likelihood ratios [2]. To calculate the sample size needed to estimate AUROC with a desired precision of the confidence interval, it is possible to resort to an online calculator [39] specifying:

 − level of confidence, (1 - α);
 − the proportion of subjects with the disease; and
 − the desired width of the confidence interval.

#### Example 8

**Fig. 9:**
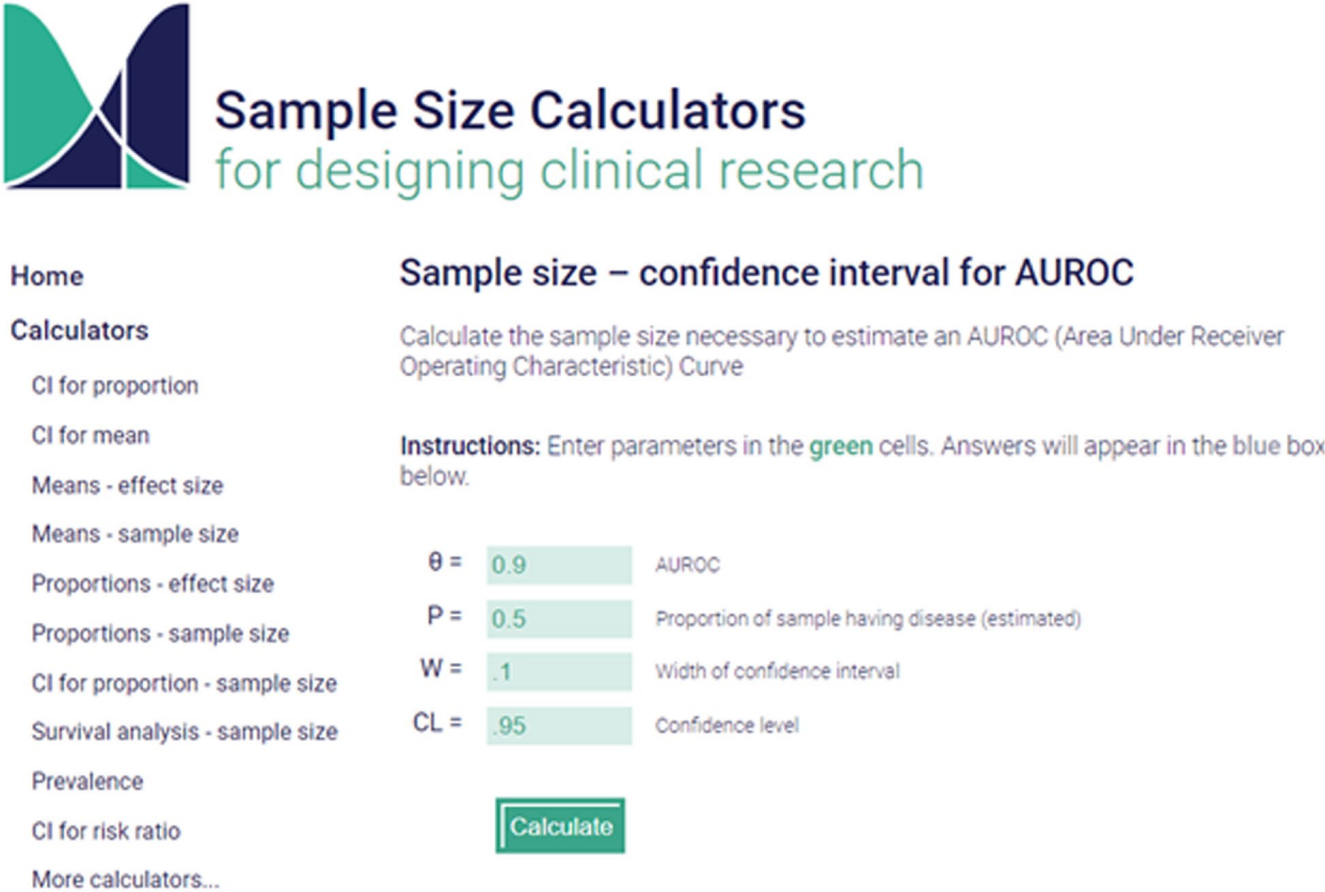
Example of sample size calculation to estimate an area under receiver operating characteristics curve (AUROC)

Suppose that the goal of the study is to estimate the AUROC, expected to be 0.9, with a 95% confidence interval length equal to 0.1 and when the prevalence in the sample is 50%. Using the calculator a sample size of 158 subjects is obtained, as per Fig. 9. The same can be obtained using the R function "prec_auc" in the package presize [40].

To calculate the sample size to detect differences between AUROCs, the following information is needed:

 − level of confidence, or desired *α* error;
 − desired statistical power, or 1 - β; and
 − the two AUROCs to be compared, θ_1_ and θ_2_.

Subsequently, the sample size can be calculated as follows [11]:

7 $$n={\left(\frac{{Z}_{\alpha }\sqrt{2{V}_{1}}+{Z}_{\upbeta }\sqrt{{V}_{1}+{V}_{2}}}{d}\right)}^{2}$$

where *n* is the required sample size, *Z*_*α*_ is the Z value corresponding to the desired *α* error, *Z*_*β*_ the Z value pertaining to the chosen statistical power, V_1_ and V_2_ can be computed as:

8 $${V}_{1}=\frac{{\theta }_{1}}{2-{\theta }_{1}}+\frac{2{{\theta }_{1}}^{2}}{1+{\theta }_{1}}-2{{\theta }_{1}}^{2}$$

9 $${V}_{2}=\frac{{\theta }_{2}}{2-{\theta }_{2}}+\frac{2{{\theta }_{2}}^{2}}{1+{\theta }_{2}}-2{{\theta }_{2}}^{2}$$

where θ_1_ and θ_2_ are the two anticipated areas under the curve, and d is equal to:

10 $$d={\theta }_{2}-{\theta }_{1}$$

#### Example 9

If the endpoint of the study is represented by the comparison of two receiver operating characteristic curves from two different diagnostic methods using the DeLong test [41], such as clinical data *versus* the combination of clinical and imaging data, to detect a certain disease, setting a desired *α* error of 0.05, corresponding to *Z*_*α*_ = 1.645, a desired statistical power for a one-sided test, due to expecting the proposed method to yield a better performance, or 1 - β, of 0.80, corresponding to *Z*_*β*_ = 0.84, and expected AUROCs, θ_1_ and θ_2_, of 0.825 and 0.9. Using Eq. 7, a sample size of 176 diseased and non-diseased subjects would be required for the study.

## Other considerations

We outlined the main methods for sample size calculation for studies involving the assessment of data reproducibility and diagnostic accuracy. Still, a few other caveats should be considered, as described in the following paragraphs.

### Dropouts or retrospective case exclusions

Some patient data which were initially included for a study, might be subsequently excluded due to various reasons, such as patients dropping out from a prospective study, or imaging datasets being not analysable due to technical reasons. As such, a certain percentage of data loss should be considered when calculating the sample size, which should be consequently increased to:

11 $${n}_{{\text{overall}}}=\frac{n}{1-d}$$

where *n*_*overall*_ is the sample size comprehensive of expected exclusions, *n* represents the original sample size and *d* the expected dropout proportion. For instance, considering an initial sample size of 75 cases, and expecting a potential dropout of 10%, or 0.10, one would potentially need to include 84 cases to finally have 75 cases for data analysis.

### Multiple endpoints (secondary *versus* exploratory)

Sample size calculations should be tailored so that the data numerosity is fit for the main endpoint of the study, namely the one statistical test that indicates the falsifiability of the null hypothesis of the study. Subsequent endpoints of the same work may or may not present with a sufficient sample size to provide a definite claim about individual null hypotheses. In particular, if the sample size is adequate for subsequent analyses, such endpoints can be deemed *secondary*. Conversely, whenever the sample size is not sufficient to reach the desired statistical power or precision, an endpoint can be deemed *exploratory*. To review whether the sample size fits further analyses, one ought to simply calculate the required sample size for each individual analysis and check whether it is lower, thus adequate, or higher. Even if the power for secondary endpoints is adequate, the conclusion about those endpoints does not have the same level of evidence of the primary endpoint due to the type 1 error inflation (see below the selection of *α* error in the case of multiple comparisons).

### Lack of prior data estimates

Especially when assessing novel techniques, prior literature data on which sample size calculations could be based might be lacking. In such instances, it may be recommended to use safe estimates, possibly opting for larger sample sizes whenever possible, to work with a data numerosity that could fit different outcomes.

### Selection of α and β values

In most cases, setting an *α* error of 0.05 and a *β* error of 0.20 may be adequate. However, such estimates should be varied considering the potential assumptions and outcomes of the study [42]. For instance, a 5% chance of wrongly observing a higher accuracy for a novel, invasive, diagnostic test may be too steep if high risks are involved. In this setting, the bar for the *α* error should probably be lowered, for instance to 0.01. Moreover, when performing multiple statistical tests altogether as primary endpoints, and the use of methods correction (such as the classical Bonferroni correction [43]) is warranted, the desired *α* error threshold ought to be adjusted accordingly when computing the required sample size for the study endpoints. This can be the case especially when a high number of comparisons are performed, as in genetics or artificial intelligence (machine/deep learning) applications [44].

## Conclusions

In conclusion, planning an appropriate sample size for a study is vital to obtain results supporting robust conclusions. When planning a study where the data pool is not too limited by external constraints, proper sample size calculations may lead to a good balance between statistical power, accuracy, and optimising workflows, leading to include the right amount of data for the specific aim of each study. This allows to avoid facing embarrassing situations such as finding a certainly clinically relevant difference in diagnostic performance between two imaging techniques (suppose 95% *versus* 85% of sensitivity) not supported by statistical significance, most likely due to the study proving underpowered.

Conversely, the risk of oversampling should also be considered. There is no reason to spend resources on working with a larger amount of data when, using a smaller, well calculated, sample size can allow to obtain statistically significant results. So, sample size calculation also respects a principle of economy, avoiding the waste of money and human resources and following important ethical principle to expose to risks no more than the required number of patients.

Of course, we should not forget the general rule that statistical significance per se is not the aim of a study. Only when the estimate of a difference or of any other parameter is both *statistically significant* and *clinically relevant* (or at least potentially clinically relevant), we will have taken a step forward, whether small or large will depending on circumstances that are often not immediately assessable. Serendipity of many medical innovations is, by definition, unpredictable.

## Data Availability

Non applicable.

## Abbreviations

AUROC: Areas under the receiver operating characteristics curve
ICC: Intraclass correlation coefficients
